# Supplementary material for: The integrated genomic and epigenomic landscape of brainstem glioma
Source: Nat Commun. 2020 Jun 17;11:3077. doi: 10.1038/s41467-020-16682-y (PMC7299931; doi:10.1038/s41467-020-16682-y)
Supplement: Supplementary file 1 — Supplementary Information [file 41467_2020_16682_MOESM1_ESM.pdf]

a

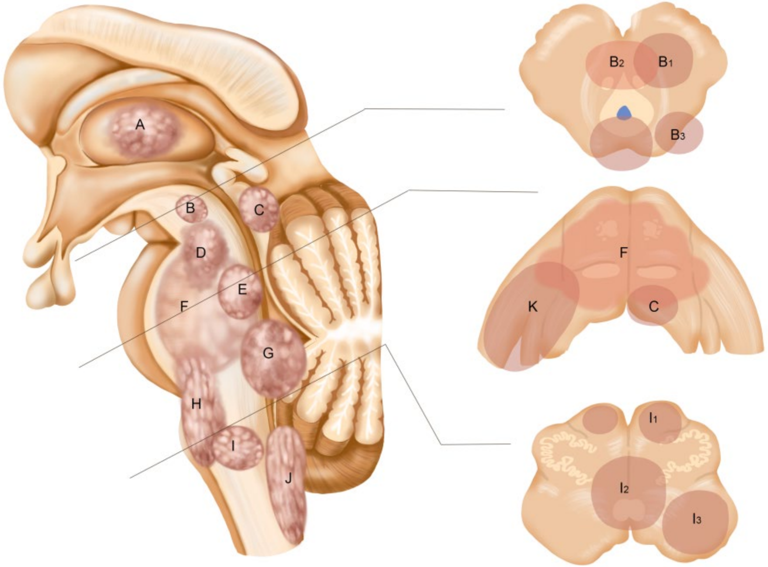

b

Code	Type and location	Number of cases
A	Midbrain-thalamus	5
B	Midbrain tegmentum	11
B1		7
B2		1
B3		3
C	Tectum	5
D	Pontomesencephalic junction	2
E	Focal pontine tumor	5
F	DIPG	33
G	Pontomedullary junction (dorsal)	8
H	Pontomedullary junction (ventral)	8
I	Focal intrinsic medullary	34
I1		8
I2		5
I3		21
J	Dorsal exophytic medllary	8
K	Middle cerebellar peduncle	7

Supplementary Figure 1 Detailed illustration of thalamus and brainstem, and summary of samples in this study (a) Locations of tumors selected for this study. These codes are explained in panel b. (b) Counts of tumors for study in different locations.

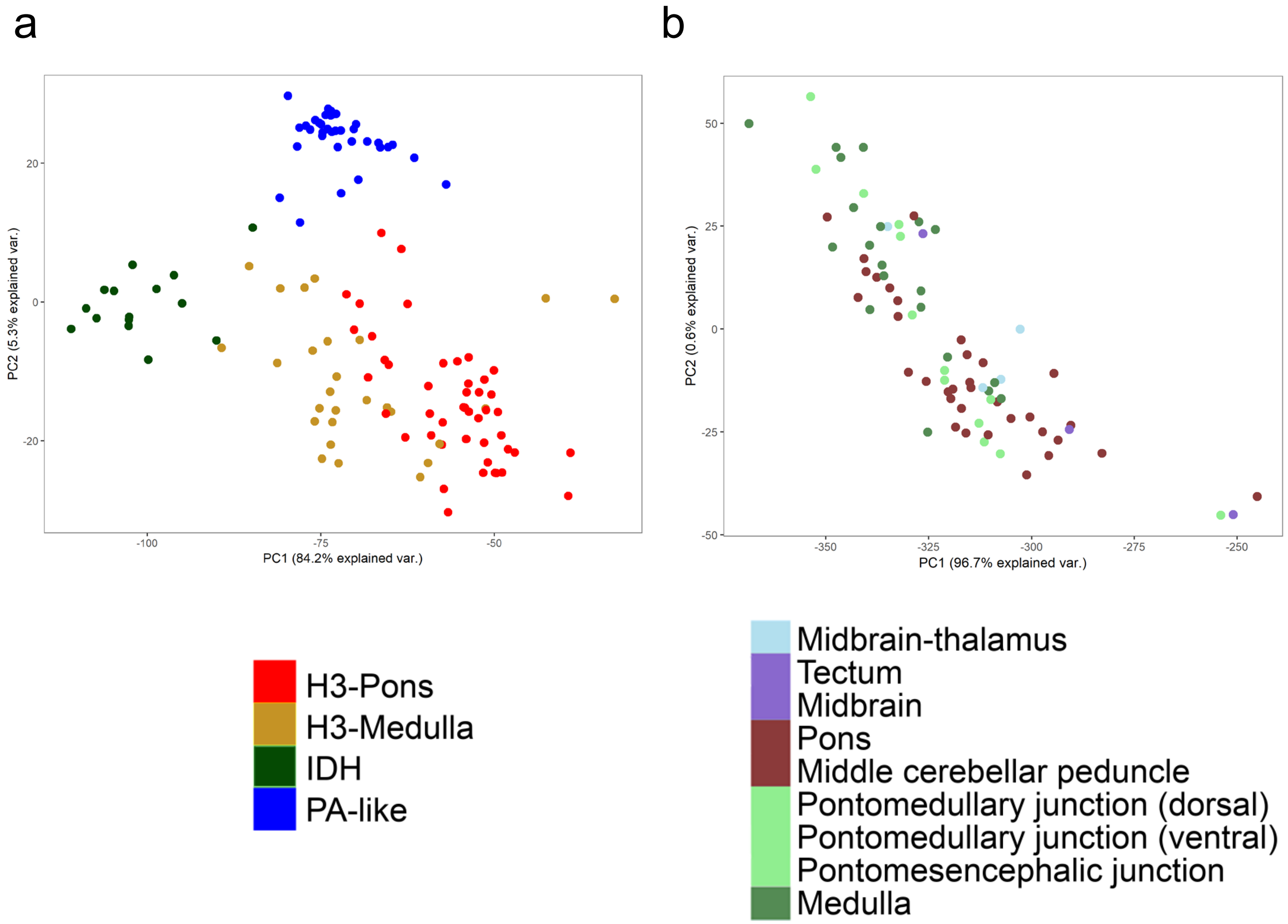

Supplementary Figure 2 Principal component analysis for tumors (a) PCA of selected probes for all tumors, colored by Methylation Clusters (b) PCA of whole probes for tumors from H3-Medulla and H3-Pons only, colored by tumor locations.

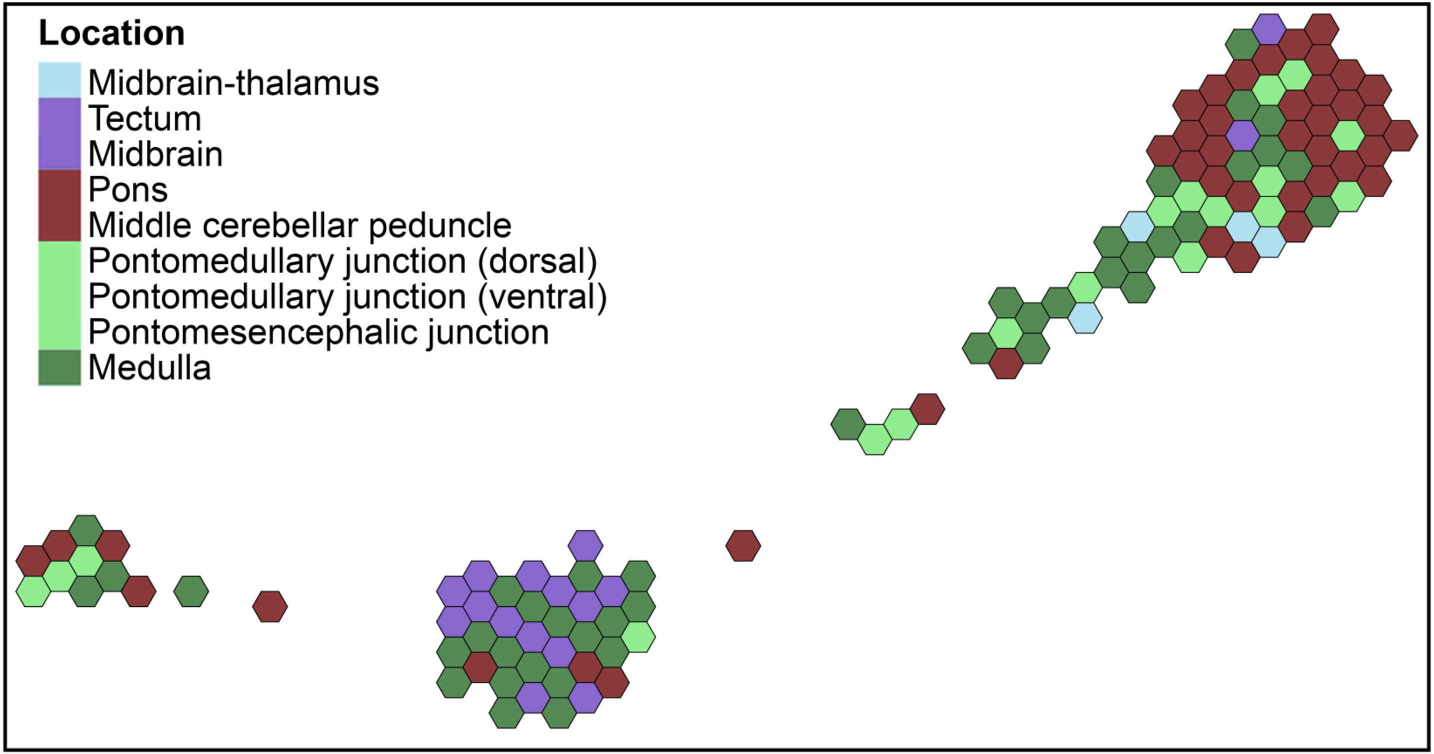

Supplementary Figure 3 Tumor map from whole probes, colored by tumor locations.

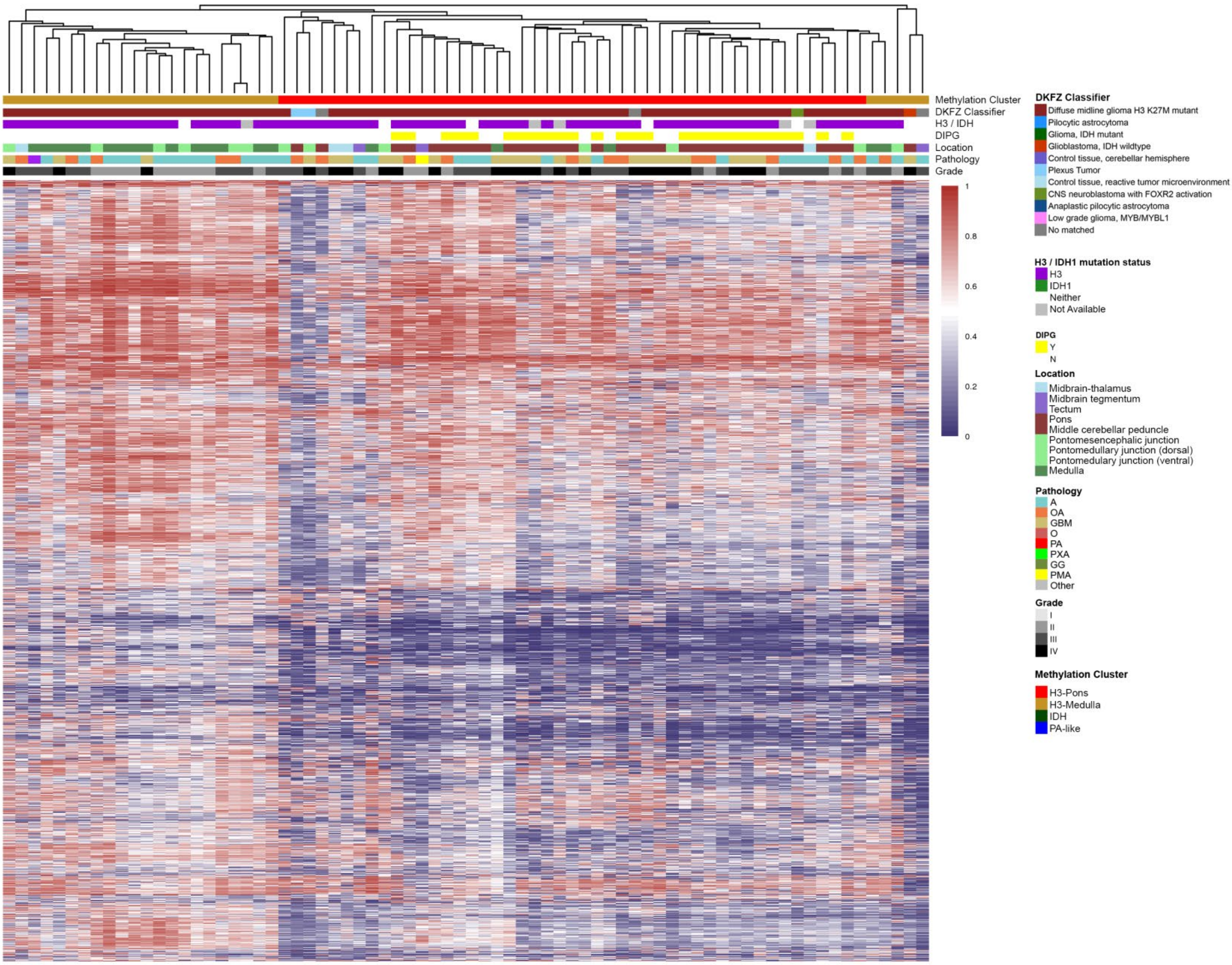

Supplementary Figure 4 Visualization of methylation status for Samples from Methylation Clusters H3-Pons and H3-Medulla only. Top 20000 variable probes across selected samples were utilized for hierarchical clustering of methylation status.

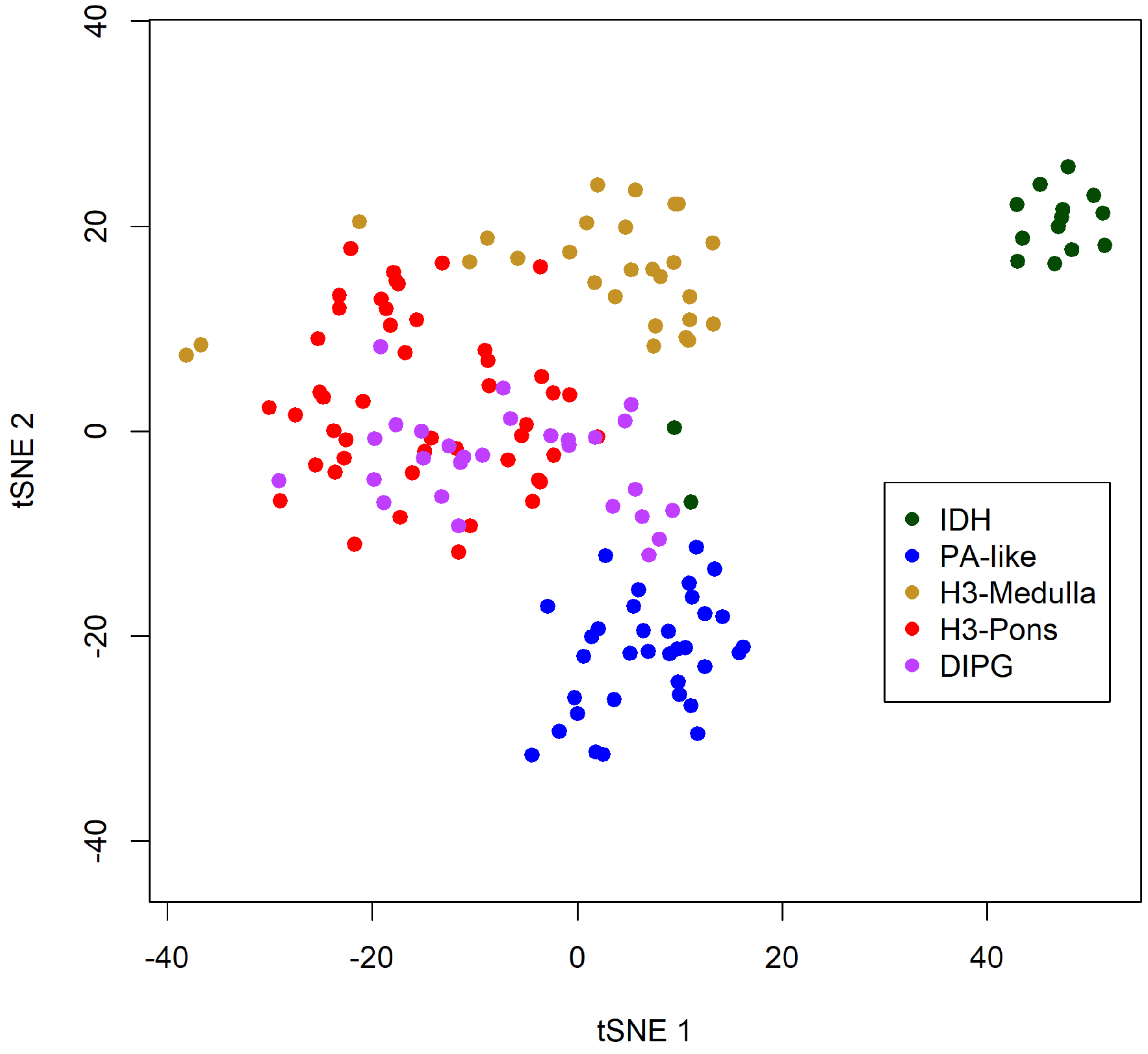

Supplementary Figure 5 Results of tSNE for top 20000 probes selected from this study. Both datasets of this study and Buczkowicz et al. are included. Dots marked as “IDH”, “PA-like”, “H3-Medulla”, and “H3-Pons” are from this study. Dots marked as “DIPG” are from dataset of Buczkowicz et al.

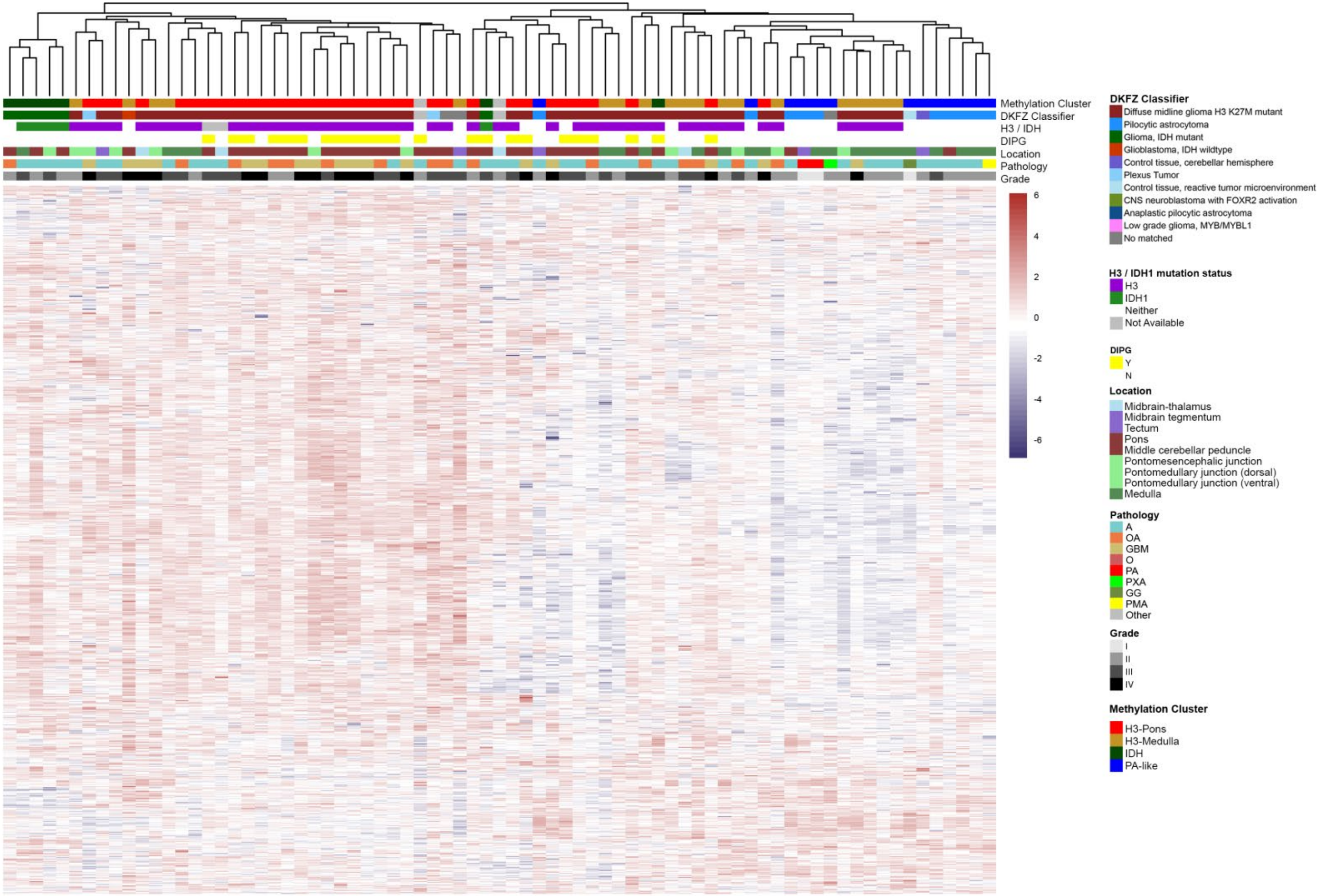

Supplementary Figure 6 Hierarchical clustering of normalized FPKM from RNAseq Genes are selected from lists of transcription factors.

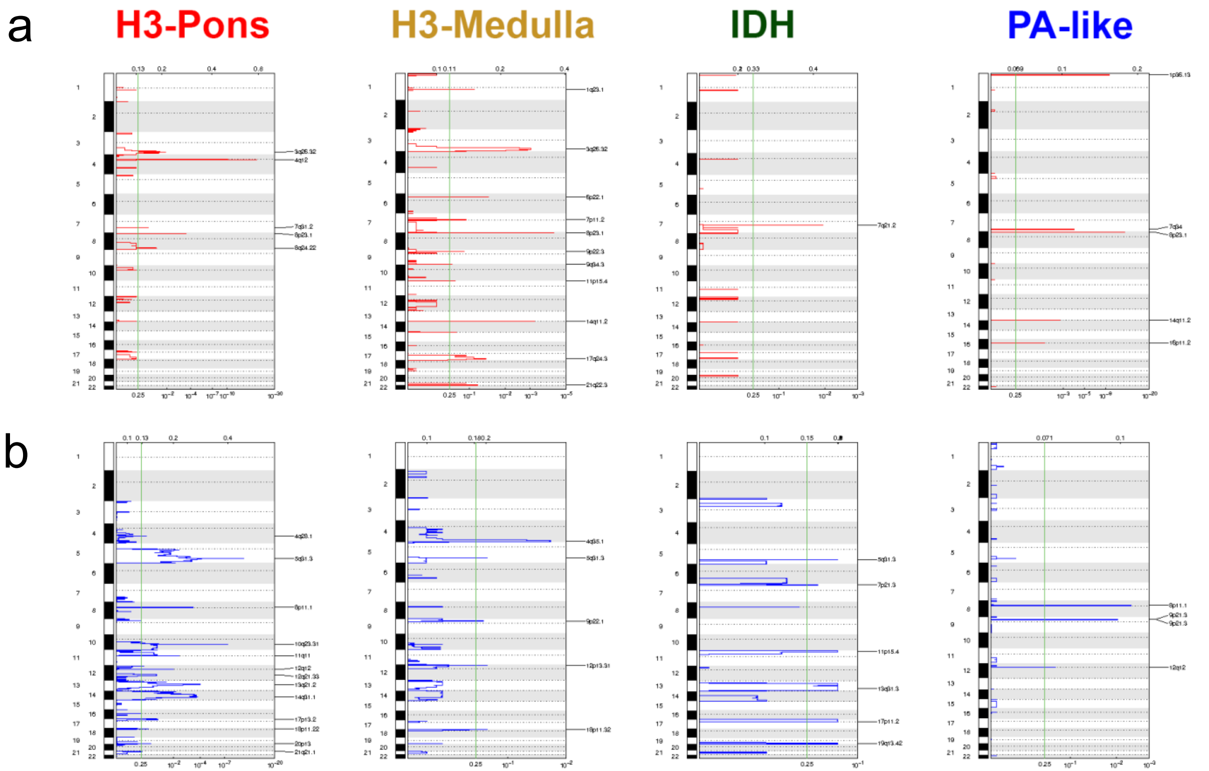

Supplementary Figure 7 Copy number alterations in four methylation clusters (a) Copy number gain (b) Copy number loss

a

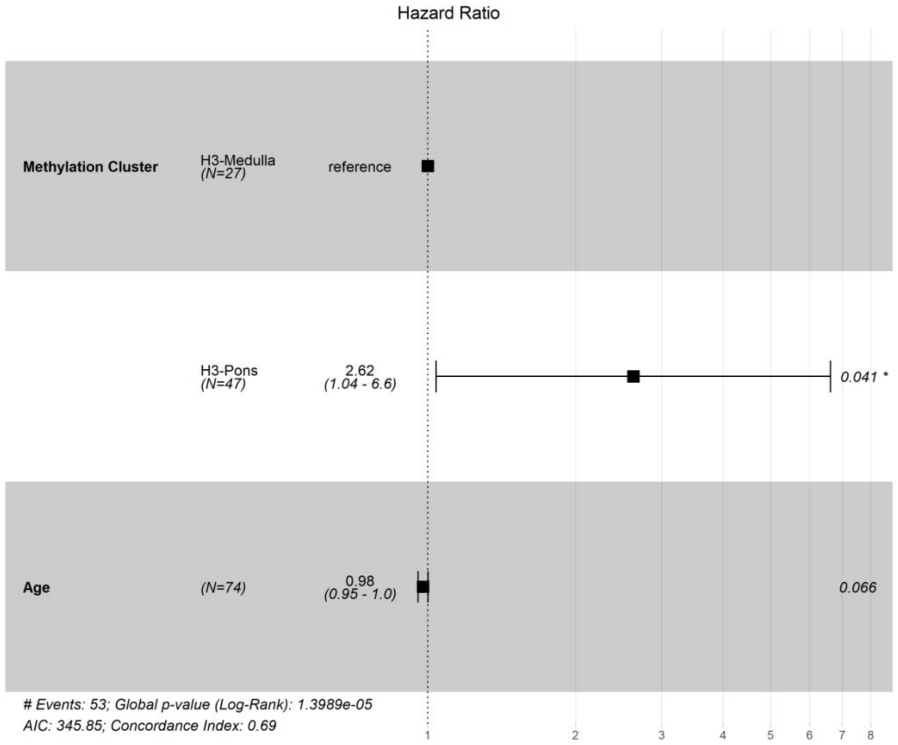

b

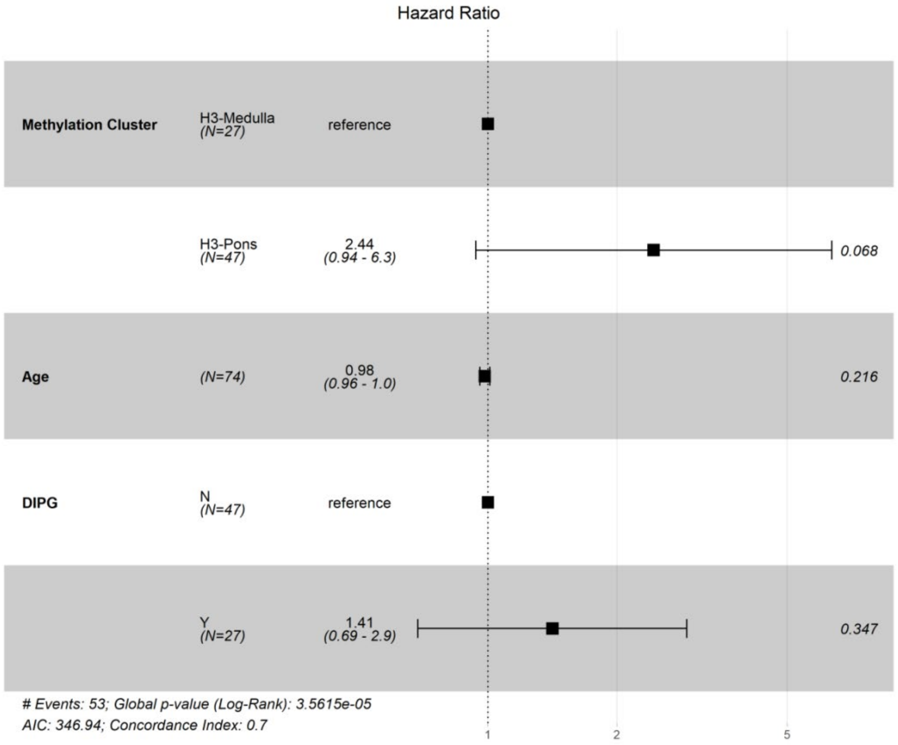

Supplementary Figure 8 Results of Cox proportional hazards regression model for multivariate survival analysis. (a) Result of regression model using the status of Methylation Cluster and Age (b) Result of regression model Including age, the diagnosis of DIPG, and status of Methylation Cluster

Supplementary Table 1 Summary table of samples, grouped by clusters defined by methylation patterns.

	**H3-Pons (n=47)**	**H3-Medulla (n=27)**	**IDH (n=15)**	**PA-like (n=34)**	**Unknown (n=3)**
**Location**					
Midbrain-thalamus	3 (6.4%)	1 (3.7%)			1 (33.3%)
Midbrain tegmentum	1 (2.1%)	1 (3.7%)	1 (6.7%)	8 (23.5%)	
Tectum	1 (2.1%)			4 (11.8%)	
Pontomesencephalic junction	2 (4.3%)				
Pons	29 (61.7%)	1 (3.7%)	3 (20.0%)	3 (8.8%)	2 (66.7%)
Pontomedullary junction (dorsal)	1 (2.1%)	6 (22.2%)	1 (6.7%)		
Pontomedullary junction (ventral)	3 (6.4%)	1 (3.7%)	3 (20.0%)	1 (2.9%)	
Medulla	3 (6.4%)	17 (63.0%)	4 (26.7%)	18 (52.9%)	
Middle cerebellar peduncle	4 (8.5%)		3 (20.0%)		
**Age**					
Median(LQ-UQ)	11 (7.0 - 24.5)	41 (26.0- 48.5)	39 (33.0-44.0)	14 (8.0-34.5)	8 (5.5-34.5)
**Survival (Months)**					
Median (CI)	9.47 (8.53 - 13.2)	26.33 (16.13 - Inf)	141.2 (24.17 - NA)	NA	8 (3.0 - Inf)
**Grade**					
I			1 (6.7%)	10 (29.4%)	
II	8 (17.0%)	11 (40.7%)	10 (66.7%)	21 (61.8%)	2 (66.7%)
III	19 (40.4%)	12 (44.4%)	4 (26.7%)	3 (8.8%)	1 (33.3%)
IV	20 (42.6%)	4 (14.8%)			
**Histology**					
A	14 (29.8%)	16 (59.3%)	9 (60.0%)	17 (50.0%)	3 (100.0%)
GBM	20 (42.6%)	4 (14.8%)			
GG				3 (8.8%)	
O		1 (3.7%)			
OA	12 (25.5%)	6 (22.2%)	5 (33.3%)	4 (11.8%)	
PA			1 (6.7%)	7 (20.6%)	
PMA	1 (2.1%)			1 (2.9%)	
PXA				1 (2.9%)	
other				1 (2.9%)	
**Gender**					
Female	25 (53.2%)	9 (33.3%)	7 (46.7%)	16 (47.1%)	2 (66.7%)
Male	22 (46.8%)	18 (66.7%)	8 (53.3%)	18 (52.9%)	1 (33.3%)
**DIPG**					
Y	27 (57.4%)		5 (33.3%)		2 (66.7%)
N	20 (42.6%)	27 (100.0%)	10 (66.7%)	34 (100.0%)	1 (33.3%)

Supplementary Table 2 Primer information for validating fusion genes

No1: Amplicon Size = 107				CAPZA2->MET
	SEQ_L2	TGGGAAGCATTCTGCTAAACTCTTTG	35	58.21
	SEQ_R2	gcggtttgtcgccagaagga	141	60.41
No2: Amplicon Size = 104				CAPZA2->MET
	SEQ_L3	TGGGAAGCATTCTGCTAAACTCTTTG	35	58.21
	SEQ_R3	ttgcagatgttcggttactgct	138	57.29
No3: Amplicon Size = 120				CAPZA2->MET
	SEQ_L2	CAGGAGCACGAGGATGCCAG	22	59.99
	SEQ_R2	gcggtttgtcgccagaagga	141	60.41
No4: Amplicon Size = 117				CAPZA2->MET
	SEQ_L2	CAGGAGCACGAGGATGCCAG	22	59.99
	SEQ_R2	ttgcagatgttcggttactgct	138	57.29
No5: Amplicon Size = 104				CAPZA2->MET
	SEQ_L2	ATTCATCACGGCGCGCTTCA	35	60.55
	SEQ_R2	ttgcagatgttcggttactgct	138	57.29
No6: Amplicon Size = 110				KMT2E->MET
	SEQ_L1	GGGGGTTGATACAGCAGAGACG	30	59.43
	SEQ_R1	caggagcacgaggatgccag	139	59.99
No7: Amplicon Size = 114				MET->CTTNBP2
	SEQ_L3	CGCCGCTGACTTCTCCACTG	43	60.54
	SEQ_R3	tctggagtgccaggaacggg	156	60.99
No8: Amplicon Size = 112				ST7,ST7,ST7,ST7,ST7,ST7,ST7,ST7,ST7->CAPZA2
	SEQ_L2	TGGTATTCATTTTCTGCAGCTTCTACA	19	57.36
	SEQ_R2	ggtctacatcctgcgggtgc	130	59.99
No9: Amplicon Size = 109				CAPZA2->CLCN1
	SEQ_L1	TCGCCAGAAGGAAGATGGCG	29	59.64
	SEQ_R1	accatcaggaaagagcatggc	137	57.54
No10: Amplicon Size = 131				CAPZA2->THAP5
	SEQ_L3	GGGAACCCATGAATCTCGCTT	11	57.27
	SEQ_R3	gcggtttgtcgccagaagga	141	60.41
No11: Amplicon Size = 100				CAPZA2->PNPLA8
	SEQ_L1	TGCTTGCTTCTCTGCTTCCCA	42	59.11
	SEQ_R1	gcggtttgtcgccagaagga	141	60.41
No12: Amplicon Size = 130				CTTNBP2->MET
	SEQ_L4	CACGAGGATGCCAGGTGCAA	28	60.13
	SEQ_R4	ggtgaacaccagtcctcgca	157	59.43
No13: Amplicon Size = 102				RP11-2B6.3->MET
	SEQ_L1	CCCCATTGCTCCTCTGCACC	14	60.2
	SEQ_R1	ctccgcgagcgcgagATAAA	115	60.13
No14: Amplicon Size = 115				SYS1-DBNDD2,DBNDD2,DBNDD2,DBNDD2,DBNDD2,DBNDD2,DBNDD2,DBNDD2,DBNDD2,DBNDD2->SYS1
	SEQ_L1	ACTGCTTTCCTCGCCGGATG	40	59.92
	SEQ_R1	ccacatgagaccacaggccc	154	59.85
No15: Amplicon Size = 105				C15orf57->CBX3
	SEQ_L1	CGCCTACAGCTCAAGCCACA	54	59.85
	SEQ_R1	ttgttgagaatgggagcatattgaca	158	57.24
