# Supplementary material for: The integrated genomic and epigenomic landscape of brainstem glioma
Source: Nat Commun. 2020 Jun 17;11:3077. doi: 10.1038/s41467-020-16682-y (PMC7299931; doi:10.1038/s41467-020-16682-y)
Supplement: Supplementary file 3 — Description of Additional Supplementary Files [file 41467_2020_16682_MOESM3_ESM.pdf]

Description of Additional Supplementary Files

File Name: Supplementary Data 1
Description: Clinical information for patients selected in this study.

File Name: Supplementary Data 2
Description: Top 20000 variable probes selected for hierarchical clustering.

File Name: Supplementary Data 3
Description: Differential expressed gens between H3-Pons and H3-Medulla identified by edgeR.

File Name: Supplementary Data 4
Description: Results of enrichment analysis by DAVID, genes selected from Supplementary Data 3.

File Name: Supplementary Data 5
Description: Results of fusion genes detected by STAR-fusion.

File Name: Supplementary Data 6
Description: Results of genomic structural variants detected by Manta.

File Name: Supplementary Data 7
Description: Results of copy number variation by GISTIC.

File Name: Supplementary Data 8
Description: Amplification/Deletion genes from GISTIC.

File Name: Supplementary Data 9
Description: Data Access Agreements for datasets deposited in BIG and EGA
