# Supplementary material for: The integrated genomic and epigenomic landscape of brainstem glioma
Source: Nat Commun. 2020 Jun 17;11:3077. doi: 10.1038/s41467-020-16682-y (PMC7299931; doi:10.1038/s41467-020-16682-y)
Supplement: Supplementary file 12 — Supplementary Data 9 [file 41467_2020_16682_MOESM12_ESM.pdf]

DATA ACCESS AGREEMENT for Whole Genome Sequencing Data in Genome Sequence Archive in BIG Data Center, Beijing Institute of Genomics (BIG)

These terms and conditions govern access to the managed access datasets (details of which are set out in Appendix I) to which the User Institution has requested access. The User Institution agrees to be bound by these terms and conditions.

Definitions

Authorised Personnel: The individuals at the User Institution to whom the IRB of Beijing Tiantan Hospital, Capital Medical University grants access to the Data. This includes the User, the individuals listed in Appendix II and any other individuals for whom the User Institution subsequently requests access to the Data. Details of the initial Authorised Personnel are set out in Appendix II.

Data: The managed access datasets to which the User Institution has requested access.

Data Producers: Dr. Liwei Zhang and the collaborators listed in Appendix I responsible for the development, organisation, and oversight of these Data.

External Collaborator: A collaborator of the User, working for an institution other than the User Institution.

Project: The project for which the User Institution has requested access to these Data. A description of the Project is set out in Appendix II.

Publications: Includes, without limitation, articles published in print journals, electronic journals, reviews, books, posters and other written and verbal presentations of research.

Research Participant: An individual whose data form part of these Data.

Research Purposes: Shall mean research that is seeking to advance the understanding of genetics and genomics, including the treatment of disorders, and work on statistical methods that may be applied to such research.

User: The principal investigator for the Project.

User Institution(s): The Institution that has requested access to the Data.

Data Producers: Department of Neurosurgery,
University of Capital Medical University, Beijing, China
Nan Si Huan Xi Lu 119, Feng Tai District, Beijing, 100070
China

1. The User Institution agrees to only use these Data for the purpose of the Project (described in Appendix II) and only for Research Purposes. The User Institution further agrees that it will only use these Data for Research Purposes which are within the limitations (if any) set out in Appendix I.

2. The User Institution agrees to preserve, at all times, the confidentiality of these Data. In particular, it undertakes not to use, or attempt to use these Data to compromise or otherwise infringe the confidentiality of information on Research Participants. Without prejudice to the generality of the foregoing, the User Institution agrees to use at least the measures set out in Appendix I to protect these Data.

3. The User Institution agrees to protect the confidentiality of Research Participants in any research papers or publications that they prepare by taking all reasonable care to limit the possibility of identification.

4. The User Institution agrees not to link or combine these Data to other information or archived data available in a way that could re-identify the Research Participants, even if access to that data has been formally granted to the User Institution or is freely available without restriction.

5. The User Institution agrees only to transfer or disclose these Data, in whole or part, or any material derived from these Data, to the Authorised Personnel. Should the User Institution wish to share these Data with an External Collaborator, the External Collaborator must complete a separate application for access to these Data.

6. The User Institution agrees that the Data Producers, and all other parties involved in the creation, funding or protection of these Data: a) make no warranty or representation, express or implied as to the accuracy, quality or comprehensiveness of these Data; b) exclude to the fullest extent permitted by law all liability for actions, claims, proceedings, demands, losses (including but not limited to loss of profit), costs, awards damages and payments made by the Recipient that may arise (whether directly or indirectly) in any way whatsoever from the Recipient's use of these Data or from the unavailability of, or break in access to, these Data for whatever reason and; c) bear no responsibility for the further analysis or interpretation of these Data.

7. The User Institution agrees to follow the *Fort Lauderdale Guidelines* (*http://www.wellcome.ac.uk/stellent/groups/corporatesite/@policy_communications/documents/web_document/wtd003207.pdf*) and the *Toronto Statement* (*http://www.nature.com/nature/journal/v461/n7261/full/461168a.html*). This includes but is not limited to recognising the contribution of the Data Producers and including a proper acknowledgement in all reports or publications resulting from the use of these Data.

8. The User Institution agrees to follow the *Publication Policy* in Appendix III. This includes respecting the moratorium period for the Data Producers to publish the first peer-reviewed report describing and analysing these Data.

9. The User Institution agrees not to make intellectual property claims on these Data and not to use intellectual property protection in ways that would prevent or block access to, or use of, any element of these Data, or conclusion drawn directly from these Data.

10. The User Institution can elect to perform further research that would add intellectual and resource capital to these data and decide to obtain intellectual property rights on these downstream discoveries. In this case, the User Institution agrees to implement licensing policies that will not obstruct further research and to follow the U.S. National Institutes of Health *Best Practices for the*

Licensing of Genomic Inventions (2005) (*https://www.icgc.org/files/daco/NIH_BestPracticesLicensingGenomicInventions_2005_en.pdf*) in conformity with the Organisation for Economic Co-operation and Development *Guidelines for the Licensing of the Genetic Inventions (2006)* (*http://www.oecd.org/science/biotech/36198812.pdf*).

11. The User Institution agrees to destroy/discard the Data held, once it is no longer used for the Project, unless obliged to retain the data for archival purposes in conformity with audit or legal requirements.

12. The User Institution will notify Dr. Liwei Zhang within 30 days of any changes or departures of Authorised Personnel.

13. The User Institution will notify Dr. Liwei Zhang prior to any significant changes to the protocol for the Project.

14. The User Institution will notify Dr. Liwei Zhang as soon as it becomes aware of a breach of the terms or conditions of this agreement.

15. Dr. Liwei Zhang may terminate this agreement by written notice to the User Institution. If this agreement terminates for any reason, the User Institution will be required to destroy any Data held, including copies and backup copies. This clause does not prevent the User Institution from retaining these data for archival purpose in conformity with audit or legal requirements.

16. The User Institution accepts that it may be necessary for the Data Producers to alter the terms of this agreement from time to time. As an example, this may include specific provisions relating to the Data required by Data Producers other than Dr. Liwei Zhang. In the event that changes are required, the Data Producers or their appointed agent will contact the User Institution to inform it of the changes and the User Institution may elect to accept the changes or terminate the agreement.

17. If requested, the User Institution will allow data security and management documentation to be inspected to verify that it is complying with the terms of this agreement.

18. The User Institution agrees to distribute a copy of these terms to the Authorised Personnel. The User Institution will procure that the Authorised Personnel comply with the terms of this agreement.

Agreed for User Institution

**Signature:**	
**Name:**	
**Title:**	
**Date:**	

Principal Investigator

I confirm that I have read and understood this Agreement.

**Signature:**	
**Name:**	
**Title:**	
**Date:**	

Agreed for data producer

**Signature:**	
**Name:**	**Liwei Zhang**
**Title:**	**Professor of Neurosurgery**
**Date:**	

APPENDIX I – DATASET DETAILS
APPENDIX II —PROJECT DETAILS
APPENDIX III — PUBLICATION POLICY

APPENDIX I – DATASET DETAILS

Dataset reference (GSA Study ID and Dataset Details)
GSA dataset ID: HRA000092
Data including whole genome sequencing data

Name of project that created the dataset

The integrated genomic and epigenomic landscape of brainstem glioma

Names of other data producers/collaborators

None

Specific limitations on areas of research

None.

Minimum protection measures required

File access: *Data can be held in unencrypted files on an institutional compute system, with Unix user group read/write access for one or more appropriate groups but not Unix world read/write access behind a secure firewall. Laptops holding these data should have password protected logins and screenlocks (set to lock after 5 min of inactivity). If held on USB keys or other portable hard drives, the data must be encrypted.*

APPENDIX II – PROJECT DETAILS (to be completed by the Requestor)

Details of dataset requested i.e., BIG Data Center Study and Dataset Accession Number

Brief abstract of the Project in which the Data will be used (500 words max)

All Individuals who the User Institution to be named as registered users

Name of Registered User	***Email***	***Job Title***	***Supervisor****

All Individuals that should have an account created at the BIG Data Center

**Name of Registered User**	**Email**	**Job Title**

APPENDIX III – PUBLICATION POLICY

The data deposition to the Genome Sequence Archive in BIG Data Center, Beijing Institute of Genomics (BIG), and the public use of the raw data have been approved by Ministry of Science and Technology of the People's Republic of China.

Dr. Liwei Zhang intend to publish the results of their analysis of this dataset and do not consider its deposition into public databases to be the equivalent of such publications. Dr. Liwei Zhang anticipate that the dataset could be useful to other qualified researchers for a variety of purposes. However, some areas of work are subject to a publication moratorium.

The publication moratorium covers any publications (including oral communications) that describe the use of the dataset. For research papers, submission for publication should not occur until 6 months after these data were first made available on the relevant hosting database, unless Dr. Liwei Zhang has provided written consent to earlier submission.

In any publications based on these data, please describe how the data can be accessed, including the name of the hosting database and its accession numbers, and acknowledge its use in a form agreed by the User Institution with Dr. Liwei Zhang.

DATA ACCESS AGREEMENT for methylation, RNAseq and targeted panel sequencing data in the European Genome-phenome Archive

These terms and conditions govern access to the managed access datasets (details of which are set out in Appendix I) to which the User Institution has requested access. The User Institution agrees to be bound by these terms and conditions.

Definitions

Authorised Personnel*:* The individuals at the User Institution to whom the IRB of Beijing Tiantan Hospital, Capital Medical University grants access to the Data. This includes the User, the individuals listed in Appendix II and any other individuals for whom the User Institution subsequently requests access to the Data. Details of the initial Authorised Personnel are set out in Appendix II.

Data*:* The managed access datasets to which the User Institution has requested access.

Data Producers*:* Dr. Liwei Zhang and the collaborators listed in Appendix I responsible for the development, organisation, and oversight of these Data.

External Collaborator*:* A collaborator of the User, working for an institution other than the User Institution.

Project*:* The project for which the User Institution has requested access to these Data. A description of the Project is set out in Appendix II.

Publications*:* Includes, without limitation, articles published in print journals, electronic journals, reviews, books, posters and other written and verbal presentations of research.

Research Participant*:* An individual whose data form part of these Data.

Research Purposes: Shall mean research that is seeking to advance the understanding of genetics and genomics, including the treatment of disorders, and work on statistical methods that may be applied to such research.

User*:* The principal investigator for the Project.

User Institution(s)*:* The Institution that has requested access to the Data.

Data Producers: Department of Neurosurgery,
University of Capital Medical University, Beijing, China
Nan Si Huan Xi Lu 119, Feng Tai District, Beijing, 100070
China

1. The User Institution agrees to only use these Data for the purpose of the Project (described in Appendix II) and only for Research Purposes. The User Institution further agrees that it will only use these Data for Research Purposes which are within the limitations (if any) set out in Appendix I.

2. The User Institution agrees to preserve, at all times, the confidentiality of these Data. In particular, it undertakes not to use, or attempt to use these Data to compromise or otherwise infringe the confidentiality of information on Research Participants. Without prejudice to the generality of the foregoing, the User Institution agrees to use at least the measures set out in Appendix I to protect these Data.

3. The User Institution agrees to protect the confidentiality of Research Participants in any research papers or publications that they prepare by taking all reasonable care to limit the possibility of identification.

4. The User Institution agrees not to link or combine these Data to other information or archived data available in a way that could re-identify the Research Participants, even if access to that data has been formally granted to the User Institution or is freely available without restriction.

5. The User Institution agrees only to transfer or disclose these Data, in whole or part, or any material derived from these Data, to the Authorised Personnel. Should the User Institution wish to share these Data with an External Collaborator, the External Collaborator must complete a separate application for access to these Data.

6. The User Institution agrees that the Data Producers, and all other parties involved in the creation, funding or protection of these Data: a) make no warranty or representation, express or implied as to the accuracy, quality or comprehensiveness of these Data; b) exclude to the fullest extent permitted by law all liability for actions, claims, proceedings, demands, losses (including but not limited to loss of profit), costs, awards damages and payments made by the Recipient that may arise (whether directly or indirectly) in any way whatsoever from the Recipient's use of these Data or from the unavailability of, or break in access to, these Data for whatever reason and; c) bear no responsibility for the further analysis or interpretation of these Data.

7. The User Institution agrees to follow the *Fort Lauderdale Guidelines* (*http://www.wellcome.ac.uk/stellent/groups/corporatesite/@policy_communications/documents/web_document/wtd003207.pdf*) and the *Toronto Statement* (*http://www.nature.com/nature/journal/v461/n7261/full/461168a.html*). This includes but is not limited to recognising the contribution of the Data Producers and including a proper acknowledgement in all reports or publications resulting from the use of these Data.

8. The User Institution agrees to follow the *Publication Policy* in Appendix III. This includes respecting the moratorium period for the Data Producers to publish the first peer-reviewed report describing and analysing these Data.

9. The User Institution agrees not to make intellectual property claims on these Data and not to use intellectual property protection in ways that would prevent or block access to, or use of, any element of these Data, or conclusion drawn directly from these Data.

10. The User Institution can elect to perform further research that would add intellectual and resource capital to these data and decide to obtain intellectual property rights on these downstream discoveries. In this case, the User Institution agrees to implement licensing policies that will not obstruct further research and to follow the U.S. National Institutes of Health *Best Practices for the*

Licensing of Genomic Inventions (2005) (https://www.icgc.org/files/daco/NIH_BestPracticesLicensingGenomicInventions_2005_en.pdf) in conformity with the Organisation for Economic Co-operation and Development *Guidelines for the Licensing of the Genetic Inventions (2006)* (http://www.oecd.org/science/biotech/36198812.pdf).

11. The User Institution agrees to destroy/discard the Data held, once it is no longer used for the Project, unless obliged to retain the data for archival purposes in conformity with audit or legal requirements.

12. The User Institution will notify Dr. Liwei Zhang within 30 days of any changes or departures of Authorised Personnel.

13. The User Institution will notify Dr. Liwei Zhang prior to any significant changes to the protocol for the Project.

14. The User Institution will notify Dr. Liwei Zhang as soon as it becomes aware of a breach of the terms or conditions of this agreement.

15. Dr. Liwei Zhang may terminate this agreement by written notice to the User Institution. If this agreement terminates for any reason, the User Institution will be required to destroy any Data held, including copies and backup copies. This clause does not prevent the User Institution from retaining these data for archival purpose in conformity with audit or legal requirements.

16. The User Institution accepts that it may be necessary for the Data Producers to alter the terms of this agreement from time to time. As an example, this may include specific provisions relating to the Data required by Data Producers other than Dr. Liwei Zhang. In the event that changes are required, the Data Producers or their appointed agent will contact the User Institution to inform it of the changes and the User Institution may elect to accept the changes or terminate the agreement.

17. If requested, the User Institution will allow data security and management documentation to be inspected to verify that it is complying with the terms of this agreement.

18. The User Institution agrees to distribute a copy of these terms to the Authorised Personnel. The User Institution will procure that the Authorised Personnel comply with the terms of this agreement.

19. This agreement (and any dispute, controversy, proceedings or claim of whatever nature arising out of this agreement or its formation) shall be construed, interpreted and governed by the laws of England and Wales and shall be subject to the exclusive jurisdiction of the English courts.

Agreed for User Institution

**Signature:**	
**Name:**	
**Title:**	
**Date:**	

Principal Investigator

I confirm that I have read and understood this Agreement.

**Signature:**	
**Name:**	
**Title:**	
**Date:**	

Agreed for data producer

**Signature:**	
**Name:**	**Liwei Zhang**
**Title:**	**Professor of Neurosurgery**
**Date:**	

APPENDIX I – DATASET DETAILS
APPENDIX II —PROJECT DETAILS
APPENDIX III — PUBLICATION POLICY

APPENDIX I – DATASET DETAILS

Dataset reference (EGA Study ID and Dataset Details)
EGA Study ID: EGAS00001004341
Dataset includes 75 samples of RNAseq data, 42 samples of targeted sequencing data, and 123 samples of methylation microarray.

Name of project that created the dataset

The integrated genomic and epigenomic landscape of brainstem glioma

Names of other data producers/collaborators

None

Specific limitations on areas of research

None.

Minimum protection measures required

File access: *Data can be held in unencrypted files on an institutional compute system, with Unix user group read/write access for one or more appropriate groups but not Unix world read/write access behind a secure firewall. Laptops holding these data should have password protected logins and screenlocks (set to lock after 5 min of inactivity). If held on USB keys or other portable hard drives, the data must be encrypted.*

APPENDIX II – PROJECT DETAILS (to be completed by the Requestor)

Details of dataset requested i.e., EGA Study and Dataset Accession Number

Brief abstract of the Project in which the Data will be used (500 words max)

All Individuals who the User Institution to be named as registered users

Name of Registered User	***Email***	***Job Title***	***Supervisor****

All Individuals that should have an account created at the EGA

**Name of Registered User**	**Email**	**Job Title**

APPENDIX III – PUBLICATION POLICY

The data deposition to the European Genome-phenome Archive and the public use of the raw data have been approved by Ministry of Science and Technology of the People's Republic of China.

Dr. Liwei Zhang intend to publish the results of their analysis of this dataset and do not consider its deposition into public databases to be the equivalent of such publications. Dr. Liwei Zhang anticipate that the dataset could be useful to other qualified researchers for a variety of purposes. However, some areas of work are subject to a publication moratorium.

The publication moratorium covers any publications (including oral communications) that describe the use of the dataset. For research papers, submission for publication should not occur until 6 months after these data were first made available on the relevant hosting database, unless Dr. Liwei Zhang has provided written consent to earlier submission.

In any publications based on these data, please describe how the data can be accessed, including the name of the hosting database (e.g., The European Genome-phenome Archive at the European Bioinformatics Institute) and its accession numbers (e.g., EGAS00000000029), and acknowledge its use in a form agreed by the User Institution with Dr. Liwei Zhang.
