# Supplementary material for: The integrated genomic and epigenomic landscape of brainstem glioma
Source: Nat Commun. 2020 Jun 17;11:3077. doi: 10.1038/s41467-020-16682-y (PMC7299931; doi:10.1038/s41467-020-16682-y)
Supplement: Supplementary file 13 — Reporting Summary [file 41467_2020_16682_MOESM13_ESM.pdf]

nature research

Corresponding author(s): Hai Yan, Liwei Zhang

Last updated by author(s): Nov 13, 2019

Reporting Summary

Nature Research wishes to improve the reproducibility of the work that we publish. This form provides structure for consistency and transparency in reporting. For further information on Nature Research policies, see Authors & Referees and the Editorial Policy Checklist.

Statistics

For all statistical analyses, confirm that the following items are present in the figure legend, table legend, main text, or Methods section.

n/a	Confirmed	
☐	☒	The exact sample size (*n*) for each experimental group/condition, given as a discrete number and unit of measurement
☒	☐	A statement on whether measurements were taken from distinct samples or whether the same sample was measured repeatedly
☒	☐	The statistical test(s) used AND whether they are one- or two-sided<br>*Only common tests should be described solely by name; describe more complex techniques in the Methods section.*
☒	☐	A description of all covariates tested
☒	☐	A description of any assumptions or corrections, such as tests of normality and adjustment for multiple comparisons
☒	☐	A full description of the statistical parameters including central tendency (e.g. means) or other basic estimates (e.g. regression coefficient) AND variation (e.g. standard deviation) or associated estimates of uncertainty (e.g. confidence intervals)
☒	☐	For null hypothesis testing, the test statistic (e.g. *F*, *t*, *r*) with confidence intervals, effect sizes, degrees of freedom and *P* value noted<br>*Give P values as exact values whenever suitable.*
☒	☐	For Bayesian analysis, information on the choice of priors and Markov chain Monte Carlo settings
☒	☐	For hierarchical and complex designs, identification of the appropriate level for tests and full reporting of outcomes
☒	☐	Estimates of effect sizes (e.g. Cohen's *d*, Pearson's *r*), indicating how they were calculated

Our web collection on statistics for biologists contains articles on many of the points above.

Software and code

Policy information about availability of computer code

Data collection	n/a
Data analysis	BWA was used for alignment and GATK mutect2 was utilized for variant calling. For RNAseq, STAR (2.5.2b) or hisat2 (2.1.0) was use for alignment, cufflinks was used for gene expression profiling, Htseq (0.9.1) and edgeR (3.12.1) was used for differential counts analysis, and GSEA (2.0) was used for gene sets analysis. GenomeStudio Methylation Module was used for data processing of methylation microarrays and quality check. Hierarchical clustering, t-distributed stochastic neighbor embedding (tSNE), and principal component analysis by R package Rtsne (0.15), and pheatmap (1.0.10). Kaplan-Meier analysis, and log-rank test, and Cox proportional hazards regression model (R package survival 2.41.3 and survminer 0.4.3) were used to test for survival analysis. Segmentation was calculated by Conumee (1.12.0) from methylation arrays. GISTIC 2.0 was used for four different methylation clusters. Manta was applied for structural variant calling from whole genome sequencing data. We also used RNAseq data for checking structural variants, and STAR-fusion was applied for RNAseq data.

For manuscripts utilizing custom algorithms or software that are central to the research but not yet described in published literature, software must be made available to editors/reviewers. We strongly encourage code deposition in a community repository (e.g. GitHub). See the Nature Research guidelines for submitting code & software for further information.

Data

Policy information about availability of data

All manuscripts must include a data availability statement. This statement should provide the following information, where applicable:

- Accession codes, unique identifiers, or web links for publicly available datasets
- A list of figures that have associated raw data
- A description of any restrictions on data availability

Methylation microarray data and raw sequencing data of this study have been submitted to Genome Sequence Archive in BIG Data Center, Beijing Institute of Genomics (BIG), Chinese Academy of Sciences, https://bigd.big.ac.cn/gsa-human, accession number PRJCA001899.

Field-specific reporting

Please select the one below that is the best fit for your research. If you are not sure, read the appropriate sections before making your selection.

☒ Life sciences ☐ Behavioural & social sciences ☐ Ecological, evolutionary & environmental sciences

For a reference copy of the document with all sections, see nature.com/documents/nr-reporting-summary-flat.pdf

Life sciences study design

All studies must disclose on these points even when the disclosure is negative.

Sample size	We collected the samples as many as possible, and used them for whole genome sequencing, methylation array, or RNAseq depending on the quality of the samples.
Data exclusions	We do not have this type of experimental design.
Replication	We do not have this type of experimental design.
Randomization	We do not have this type of experimental design.
Blinding	We do not have this type of experimental design.

Reporting for specific materials, systems and methods

We require information from authors about some types of materials, experimental systems and methods used in many studies. Here, indicate whether each material, system or method listed is relevant to your study. If you are not sure if a list item applies to your research, read the appropriate section before selecting a response.

Materials & experimental systems

n/a	Involved in the study	
☒	☐	Antibodies
☒	☐	Eukaryotic cell lines
☒	☐	Palaeontology
☒	☐	Animals and other organisms
☒	☐	Human research participants
☒	☐	Clinical data

Methods

n/a	Involved in the study	
☒	☐	ChIP-seq
☒	☐	Flow cytometry
☒	☐	MRI-based neuroimaging
